# SGK1 Attenuates Oxidative Stress-Induced Renal Tubular Epithelial Cell Injury by Regulating Mitochondrial Function

**DOI:** 10.1155/2019/2013594

**Published:** 2019-09-18

**Authors:** Daofang Jiang, Chensheng Fu, Jing Xiao, Zhenxing Zhang, Jianan Zou, Zhibin Ye, Xiaoli Zhang

**Affiliations:** ^1^Department of Nephrology, Huadong Hospital, Fudan University, Shanghai, China; ^2^Shanghai Key Laboratory of Clinical Geriatric Medicine, Shanghai, China

## Abstract

Mitochondrial dysfunction has been implicated in the early stages or progression of many renal diseases. Improving mitochondrial function and homeostasis has the potential to protect renal function. Serum- and glucocorticoid-induced kinase 1 (SGK1) is known to regulate various cellular processes, including cell survival. In this study, we intend to demonstrate the effect and molecular mechanisms of SGK1 in renal tubular cells upon oxidative stress injury and to determine whether regulation of mitochondrial function is implicated in this process. HK-2 cells were exposed to H_2_O_2_, and cell viability and apoptosis were dynamically detected by the CCK-8 assay and annexin-V/PI staining. The concentrations of cellular reactive oxygen species (ROS) and adenosine triphosphate (ATP) and the expression of the SGK1/GSK3*β*/PGC-1*α* signaling pathway were analyzed by flow cytometry or western blot. In addition, shRNA targeting SGK1 and SB216763 were added into the culture medium before H_2_O_2_ exposure to downregulate SGK1 and GSK3*β*, respectively. Cell viability and mitochondrial functions, including mitochondrial membrane potential (*Δψ*m), Cytochrome C release, mtDNA copy number, and mitochondrial biogenesis, were examined. Protein levels and SGK1 activation were significantly stimulated by H_2_O_2_ exposure. HK-2 cells with SGK1 inhibition were much more sensitive to H_2_O_2_-induced oxidative stress injury than control group cells, as they exhibited increased apoptotic cell death and mitochondrial dysfunction involving the deterioration of cellular ATP production, ROS accumulation, mitochondrial membrane potential reduction, and release of Cytochrome C into the cytoplasm. Studies on SGK1 knockdown also indicated that SGK1 is required for the induction of proteins associated with mitochondrial biogenesis, including PGC-1*α*, NRF-1, and TFAM. Moreover, the deleterious effects of SGK1 suppression on cell apoptosis and mitochondrial function, including mitochondrial biogenesis, were related to the phosphorylation of GSK3*β* and partially reversed by SB216763 treatment. H_2_O_2_ leads to SGK1 overexpression in HK-2 cells, which protects human renal tubule cells from oxidative stress injury by improving mitochondrial function and inactivating GSK3*β*.

## 1. Introduction

Oxidative stress, characterized by excessive levels of reactive oxygen species (ROS) resulting from an imbalance between the oxidative and antioxidative systems, has been widely implicated in renal pathological conditions [1, 2]. The kidney is one of the most energy-consuming organs in the human body [3]. Owing to their active reabsorption and secretion function, renal proximal tubular cells contain the second highest mitochondrial content after only cardiomyocytes [4]. Mitochondrial dysfunction leads to decreased ATP production, increased ROS levels and, thus, loss of renal function [5, 6]. Growing evidence show that aging is a major contributor to the increasing incidence of kidney disease. Mitochondrial dysfunction is one of the most crucial hallmarks that contribute to the process of aging. So, targeting mitochondrial function and homeostasis is a potential strategy to protect renal function and retard senescence [7].

The abundance and functional properties of mitochondria are finely modulated to meet cellular metabolic and energetic demands. Mitochondrial homeostasis requires a balance among mitochondrial biogenesis, fission and fusion, and mitophagy [8]. The regulation of mitochondrial homeostasis may be achieved through a set of transcription factors that transforms environmental stimuli into cellular adaptive responses. The decreased efficiency of mitochondrial biogenesis has been evidenced in multiple renal diseases [9, 10], and restoring mitochondrial biogenesis ameliorates the development and progression of acute kidney injury (AKI) [11, 12]. However, the upstream regulatory signal for mitochondrial turnover in the setting of renal oxidative stress injury remains unknown [13].

Serum- and glucocorticoid-induced kinase 1 (SGK1) is a serine/threonine kinase that can be transcriptionally regulated by glucocorticoids, serum, and various cell stress stimuli, including oxidative stress, and it functions as a strong antiapoptotic kinase in these conditions [14]. Increased SGK1 expression and activity has been reported to protect endothelial cells against oxidative stress [15], while inhibition of SGK1 results in increased neurocyte stress injury [16, 17]. Recent studies have shown that SGK1 may influence longevity and stress resistance, underscoring the clinical relevance of SGK1 in the PI3K cascade [18–20]. Our previous study demonstrated that SGK1 is dynamically overexpressed and activated upon ischemia/reperfusion injury in rat kidneys and justified that SGK1 protects renal cells from hypoxia/reoxygenation injury via promoting autophagy [21].

Thus, we wanted to further investigate whether SGK1 is implicated in the regulation of mitochondrial function in renal tubular cells under oxidative stress injury and examine the potential molecular mechanism.

## 2. Materials and Methods

### 2.1. Cell Culture and Treatment

Human proximal tubular epithelial cells (HK-2) were purchased from American Type Culture Collection (ATCC, Manassas, VA, USA) and cultured in Dulbecco's Modified Eagle's Medium/F12 (DMEM/F12) (Thermo Fisher Scientific, Waltham, MA, USA) supplemented with 10% heat-inactivated fetal bovine serum (FBS; Thermo Fisher Scientific), 2 mmol/l glutamine, 100 U/ml penicillin, and 100 mg/ml streptomycin at 37°C in a humidified incubator containing 5% CO_2_.

Oxidative stress was induced by incubation of the HK-2 cells with hydrogen peroxide (H_2_O_2_). Briefly, after the HK-2 cells were seeded into 6- or 96-well plates, they were exposed to H_2_O_2_ at various concentrations for different amounts of time. HK-2 cells maintained in medium without H_2_O_2_ exposure were used as the normal control. For HK-2 cell transfection with shRNA, SGK1 or scramble shRNAs in the pGLV3 lentiviral vector were synthesized by GenePharma Corporation (Shanghai, China) and transfected into the HK-2 cells as described previously [21]. Briefly, the HK-2 cells were seeded in 6-well plates with serum-free DMEM and then subjected to a mixture of shRNA and Lipofectamine 3000 (Thermo Fisher Scientific) reagent. After incubation for 72 h, the medium was changed and the cells were harvested for further experiments. For the glycogen synthase kinase 3*β* (GSK3*β*) inhibitor treatment, HK-2 cells were treated with 10 *μ*mol/l (*μ*M) SB216763 (Selleck, Houston, TX, USA) 1 h prior to H_2_O_2_ exposure.

### 2.2. Cell Viability Analysis

Cell Counting Kit-8 (CCK-8; Dojindo, Kumamoto, Japan) was used to assess the viability of HK-2 cells. Briefly, 10 *μ*l of CCK-8 reagent was added to HK-2 cells, which were seeded in a 96-well plate in a humidified 5% CO_2_ atmosphere at 37°C for 3 h. The optical density (OD) was measured with a microplate reader (Thermo Fisher Scientific) at 450 nm.

### 2.3. Flow Cytometry Analysis of Apoptosis

The annexin-V/PI apoptosis assay kit (Thermo Fisher Scientific) was used according to the manufacturer's recommendation to examine the apoptotic fraction of HK-2 cells after exposure to H_2_O_2_ for the indicated times. HK-2 cells were washed twice with phosphate-buffered saline (PBS) and resuspended in 100 *μ*l of 1x binding buffer mixed with 5 *μ*l of annexin-V-FITC and 5 *μ*l of a PI staining solution for 15 min in the dark at room temperature. After 15 min of incubation, another 400 *μ*l of binding buffer was added, and the cells were analyzed using a FACSCalibur flow cytometer (BD Biosciences, Franklin Lakes, NJ, USA). Ten thousand cells from the sample were scanned, and the data were analyzed using CellQuest software (BD Biosciences).

### 2.4. Mitochondria/Cytosol Fractionation

This assay was conducted using a Mitochondria Isolation Kit (Pierce, Rockford, IL, USA) according to the manufacturer's protocol. Cells were homogenized and the homogenates were added with 1.0 ml of 1x Cytosol Extraction Buffer Mix and centrifuged at 750 × g for 10 min at 4°C. Collect the supernatant carefully and discard the pellet. Then, the supernatant was centrifuged at 12,000 × g for 15 min at 4°C; collect the supernatant and save the pellet. Next, the supernatant was further centrifuged at 100,000 × g for 1 h and the supernatant was kept as the cytosol fraction, while the pellet was resuspended with 100 *μ*l of Mitochondrial Extraction Buffer Mix and saved as a mitochondrial fraction. Then, Cytochrome C in the cytoplasm or mitochondria was detected by immunoblot analysis.

### 2.5. Western Blot Analysis

At the end of the culture period, HK-2 cells were collected and lysed with RIPA buffer supplemented with complete protease inhibitor cocktail tablets. Protein concentrations were determined with the BCA Protein Assay Kit (Thermo Fisher Scientific), and protein lysates (30-50 *μ*g) were mixed with (4x) NuPAGE LDS sample buffer, heated for 5 min at 99°C, and subjected to electrophoresis on 4%-15% polyacrylamide gradient SDS gels. The proteins were then transferred onto PVDF membranes (Millipore, Billerica, MA, USA) and incubated with antibodies against SGK1, phosphorylated-SGK1 (p-SGK1), GSK3*β*, phosphorylated-GSK3*β* (p-GSK3*β*), cleaved caspase-3, and COX IV (Cell Signaling Technology, Danvers, MA, USA); peroxisome proliferator-activator *γ* coactivator-1*α* (PGC-1*α*), Bcl-2, Bax, nuclear transcription factor-1 (NRF-1), and mitochondrial transcription factor A (TFAM) (Abcam, Cambridge, UK); and Cytochrome C and GAPDH (Sigma-Aldrich, St. Louis, MO, USA). Immunoblots were developed using an HRP-conjugated anti-rabbit or anti-mouse IgG antibody (Jackson, West Grove, PA, USA). Relative concentrations were assessed by densitometry analysis of digitized autographic images using ImageJ software.

### 2.6. Measurement of Adenosine Triphosphate (ATP) Levels

The intracellular ATP level was measured using a luciferin-luciferase assay kit (Beyotime, China) according to the manufacturer's instructions. Briefly, 40 *μ*l of cell extracts or ATP standard reaction solutions, ranging from 100 nM to 5 *μ*M, was added to 96-well luminescence assay plates. Then, 100 *μ*l of reaction buffer was added to each well. Luminescence was measured with a fluorescence microplate reader (Thermo Fisher Scientific) at a 562 nm absorbance. The cellular ATP content was calculated according to the ATP standard curve.

### 2.7. Measurement of Reactive Oxygen Species

The cellular production of ROS was measured using an ROS detection kit (Beyotime) according to the manufacturer's instructions. Briefly, HK-2 cells were incubated with 10 *μ*M DCFH-DA for 30 min in a dark environment at 37°C, washed three times with warm serum-free DMEM, trypsinized, and resuspended in 1 ml of PBS. Fluorescence was analyzed by a FACSCalibur flow cytometer (BD Biosciences).

### 2.8. Mitochondrial Membrane Potential Assay

Mitochondrial membrane potential (*ΔΨ*m) was measured using a fluorescent cationic dye, 5,5′,6,6′-tetrachloro-1,1′,3,3′-tetraethyl-imidacarbocyanine iodide (JC-1; Beyotime). The changes in *ΔΨ*m were determined by the levels of relative fluorescence units using a FACSCalibur flow cytometer (BD Biosciences) with a 488 nm excitation filter and a 525–595 nm emission filter.

### 2.9. Analysis of Mitochondrial DNA (mtDNA)

Total genomic DNA was extracted using a GenElute mammalian genomic DNA kit (Sigma-Aldrich) according to the manufacturer's instructions. ND1 was used as a mtDNA marker, and the nuclear intron of 18S RNA was used as a nDNA marker. The ND1 primers were designed from regions of the mtDNA that are not found in nuclear-encoded mitochondrial pseudogenes. The primers were as follows: ND1 forward 5′-CCTCACTCATTTACACCAACCAC-3′, reverse 5′-TATAATCACTGTGCCCGCTCA-3′; 18S RNA forward 5′-GCGGTTCTATTTTGTTGGTTTT-3′, reverse 5′-ACCTCCGACTTTCGTTCTTG-3′. The quantification of the mtDNA product was analyzed after normalization to 18S RNA.

### 2.10. Immunofluorescence Colocalization Analysis

HK-2 cells were incubated with 100 nM MitoTracker Red CMXRos (Thermo Fisher Scientific) for 30 min at 37°C to label the mitochondria. To examine the Cytochrome C location, the cells were fixed in 4% paraformaldehyde for 15 min and permeabilized with 0.1% Triton X-100 for 15 min. After blocking with 10% bovine serum albumin for 20 min at room temperature, the cells were incubated with a primary antibody against Cytochrome C (1 : 100, Sigma-Aldrich) overnight at 4°C and subsequently incubated with FITC-conjugated secondary antibodies (1 : 500, Sigma-Aldrich). The colocalization of mitochondria and Cytochrome C was visualized by a confocal microscopy system (Leica, Wetzlar, Germany).

### 2.11. Statistical Analysis

All data are presented as the mean ± standard deviation (SD). Statistical analysis was performed using Student's *t*-test or one-way ANOVA with GraphPad Prism 5.0. *P* < 0.05 was recognized as statistically significant.

## 3. Results

### 3.1. Hydrogen Peroxide Dynamically Induces HK-2 Cell Injury and Mitochondrial Dysfunction

**Figure 1:**
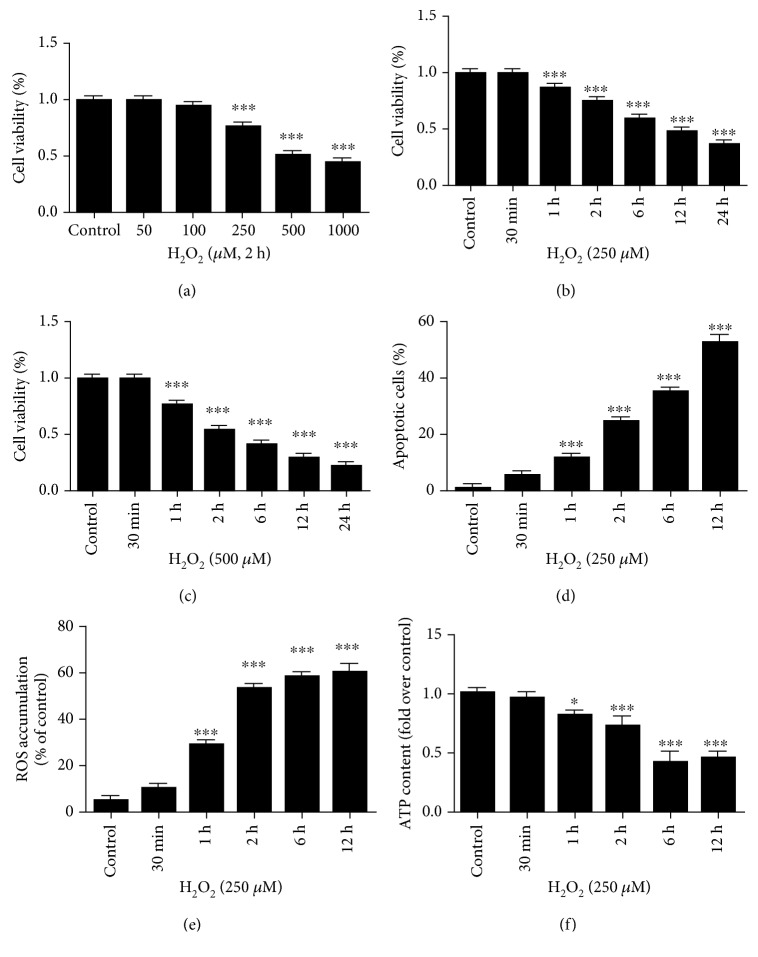
Oxidative stress induces HK-2 cell damage and mitochondrial dysfunction in a time- and dose-dependent manner. (a–c) Cell viability was measured in HK-2 cells treated with increasing doses of hydrogen peroxide (H_2_O_2_) for different time periods. (d) Cell apoptosis was determined by flow cytometric analysis with annexin-V/PI double-staining. (e) The level of cellular ROS concentration was measured via DCFHDA fluorescence by flow cytometry. (f) A luminescence assay was used to measure the cellular ATP levels. Data are presented as the mean ± SD (*n* = 3). ^∗^*P* < 0.05 and ^∗∗∗^*P* < 0.001 vs. control.

To mimic oxidative damage, we investigated the effect of different durations and doses of H_2_O_2_ exposure on the viability, apoptosis, and mitochondrial function of HK-2 cells. As expected, a 2 h treatment of the HK-2 cells with H_2_O_2_ resulted in a dose-dependent reduction in cell survival, as evidenced by the significant decrease in the number of viable cells in comparison with that in the untreated control group, especially when the concentration of H_2_O_2_ was greater than 250 *μ*M (Figure 1(a)). In addition, studies on the effects of 250 *μ*M and 500 *μ*M H_2_O_2_ exposure over time showed that H_2_O_2_ time-dependently caused HK-2 cell death (Figures 1(b) and 1(c)). Similarly, H_2_O_2_ increased HK-2 cell apoptosis in a time-dependent manner (Figure 1(d)). Moreover, in agreement with the CCK-8 assay results, in which the cell viability was approximately 75% when the HK-2 cells were treated with 250 *μ*M H_2_O_2_ for 2 h, the annexin-V/PI apoptosis examination demonstrated that the proportion of apoptotic cells under the same conditions was approximately 25%.

Consistent with the above results, a significant decline in mitochondrial function was concomitantly observed in the HK-2 cells with increasing H_2_O_2_ incubation time. The oxidant-induced cellular injury was associated with increased ROS accumulation and reduced cellular ATP levels compared with those in the control group when the HK-2 cells were exposed to H_2_O_2_ for more than 1 h (Figures 1(e) and 1(f)).

### 3.2. H_2_O_2_ Time-Dependently Stimulates the SGK1-Dependent Signaling Pathway in HK-2 Cells

**Figure 2:**
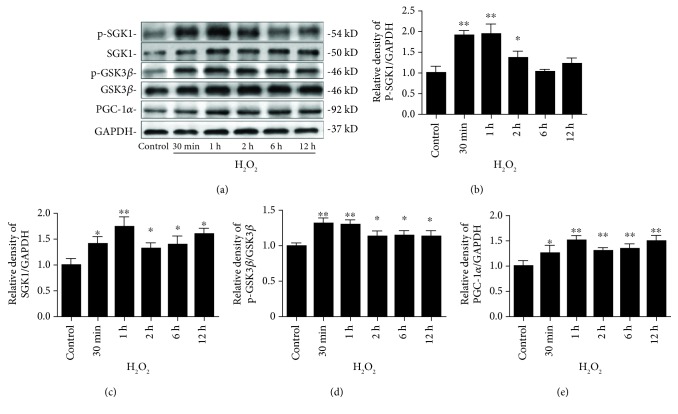
H_2_O_2_ time-dependently stimulates the SGK1-dependent signaling pathway in HK-2 cells. HK-2 cells were treated with 250 *μ*M H_2_O_2_ for different time periods. (a) Representative western blot bands are shown at the top of the corresponding graphs. (b–e) Phosphorylated SGK1 (b), total SGK1 (c), phosphorylated GSK3*β* (d), and PGC-1*α* (e) protein levels were examined by western blot analysis. Relative protein levels were normalized to GAPDH and total GSK3*β* protein levels. Data are presented as the mean ± SD (*n* = 3). ^∗^*P* < 0.05 and ^∗∗^*P* < 0.01 vs. control.

To further explore the mechanism of renal oxidative stress injury, we examined the SGK1/GSK3*β*/PGC-1*α* pathway upon oxidative stress by western blot (Figure 2(a)). Treating HK-2 cells with 250 *μ*M H_2_O_2_ induced robust cellular SGK1 activation (Figure 2(b)). Interestingly, this oxidative stress-induced SGK1 phosphorylation response in the HK-2 cells was fast, peaking within 1 h and then returning to baseline after 6 h (Figure 2(b)). The treatment of HK-2 cells with H_2_O_2_ increased SGK1 protein levels after 30 min of incubation, and these levels remained elevated until 12 h after H_2_O_2_ exposure (Figure 2(c)). These results demonstrate that H_2_O_2_ exposure promotes both the expression and activation of the SGK1 protein in HK-2 cells. Previous studies have shown that SGK1 induced by numerous stimuli confers resistance to apoptosis through multiple signaling pathways and that GSK3*β* is an important downstream target of SGK1 [22]. Thus, we sought to explore whether GSK3*β* is involved in the H_2_O_2_-induced regulation of the SGK1 pathway by measuring the phosphorylated and total levels of GSK3*β* after H_2_O_2_ treatment. Correlating with the increasing levels of SGK1, H_2_O_2_ increased the phosphorylation of GSK3*β* (Figure 2(d)). These findings indicate that upon oxidative stress, SGK1 may contribute to cell survival by phosphorylating and inactivating GSK3*β*. When the HK-2 cells were incubated with 250 *μ*M H_2_O_2_, the level of PGC-1*α* protein expression increased in a time-dependent manner (Figure 2(e)).

### 3.3. SGK1 Promotes Cell Viability and Inhibits the Apoptosis of HK-2 Cells Exposed to Oxidative Stress

**Figure 3:**
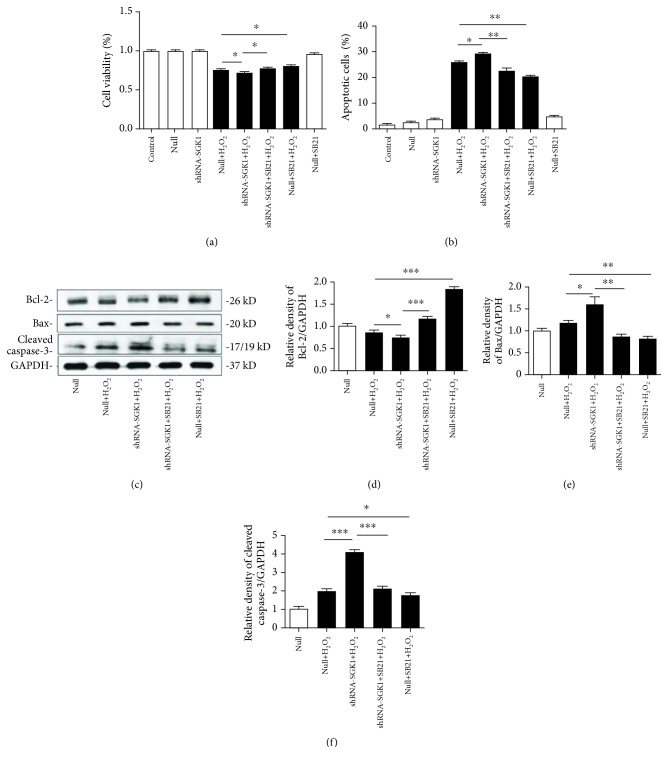
SGK1 knockdown increases H_2_O_2_-induced cell injury through the GSK3*β* pathway in HK-2 cells. HK-2 cells transfected with scramble control (null) or SGK1 shRNA (shRNA-SGK1) for 72 h were incubated with the GSK3*β* inhibitor SB216763 (SB21, 10 *μ*M) for 1 h and then treated with H_2_O_2_ (250 *μ*M) for 2 h. (a) Cell viability was assessed via the CCK-8 assay. (b) Cell apoptosis was determined by flow cytometric analysis with annexin-V/PI double-staining. (c–f) The expression levels of apoptosis-related proteins in HK-2 cells were detected by western blot analysis. Data are presented as the mean ± SD (*n* = 3). ^∗^*P* < 0.05, ^∗∗^*P* < 0.01, and ^∗∗∗^*P* < 0.001.

To further study the function of the H_2_O_2_-induced expression and phosphorylation of the SGK1 protein, we mediated the knockdown of SGK1 with shRNA and pharmacologically inhibited GSK3*β* with SB21. Specifically, HK-2 cells transfected with the scramble control (null) or SGK1 shRNA (shRNA-SGK1) for 72 h were incubated with SB21 or DMSO for 1 h and then treated with H_2_O_2_ for 2 h. After these treatments, cell viability, apoptosis, and mitochondrial function were determined. Transfection of the HK-2 cells with shRNA-SGK1 resulted in a significant reduction in cell viability (Figure 3(a)). The HK-2 cells with SGK1 inhibition had a higher apoptotic ratio under oxidative stress (Figure 3(b)). These results indicate a critical role of SGK1 in H_2_O_2_-induced oxidative injury. However, SGK1 knockdown did not influence cell viability or cell apoptosis relative to the respective control cells in normal culture conditions. We also found that apoptosis-related gene expression was affected after SGK1 regulation. At the protein level, inhibiting SGK1 significantly decreased the expression of the antiapoptotic gene Bcl-2 and distinctly increased the expression of the proapoptotic genes Bax and cleaved caspase-3 (Figures 3(d)–3(f)). Moreover, SB21 effectively ameliorated H_2_O_2_-induced HK-2 cell damage (Figures 3(a) and 3(b)) and significantly attenuated the exacerbating effects of SGK1 inhibition on cell viability and apoptosis (Figures 3(a) and 3(b)). Similarly, SB21 improved the level of Bcl-2 and further reduced the levels of Bax and cleaved caspase-3, under oxidative stress with or without SGK1 inhibition (Figures 3(d)–3(f)).

### 3.4. SGK1 Preserves Mitochondrial Function in HK-2 Cells Exposed to Oxidative Stress

**Figure 4:**
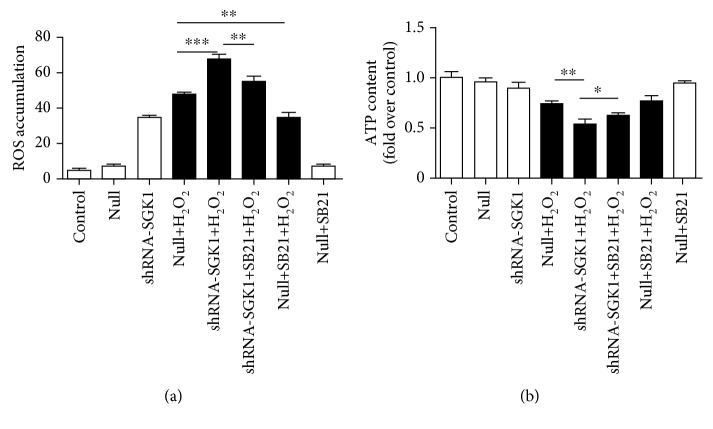
The SGK1 signaling pathway is implicated in regulating cellular ATP and ROS production upon oxidative stress. HK-2 cells transfected with scramble control (null) or SGK1 shRNA (shRNA-SGK1) for 72 h were incubated with 10 *μ*M SB21 for 1 h and then treated with or without H_2_O_2_ (250 *μ*M) for 2 h. (a) The level of cellular ROS was measured via DCFHDA fluorescence by flow cytometry. (b) A luminescence assay was used to measure the cellular ATP levels. Data are presented as the mean ± SD (*n* = 3). ^∗^*P* < 0.05, ^∗∗^*P* < 0.01, and ^∗∗∗^*P* < 0.001.

**Figure 5:**
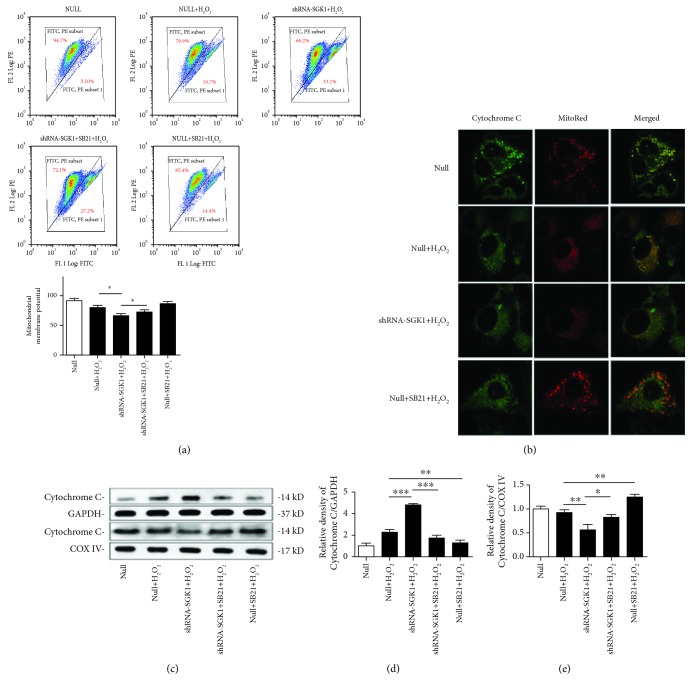
The SGK1-dependent signaling pathway modulates the mitochondrial function of HK-2 cells upon oxidative stress. HK-2 cells transfected with scramble control (null) or SGK1 shRNA (shRNA-SGK1) for 72 h were incubated with 10 *μ*M SB21 for 1 h and then treated with H_2_O_2_ (250 *μ*M) for 2 h. (a) The mitochondrial membrane potential (*ΔΨ*m) of the HK-2 cells was measured using the lipophilic cationic dye JC-1. (b) Cytochrome C release was examined by confocal microscopy in MitoTracker Red-labeled HK-2 cells. (c–e) The expression levels of Cytochrome C in the cytosolic and mitochondrial fractions of HK-2 cells were detected by western blot analysis. Data are presented as the mean ± SD (*n* = 3). ^∗^*P* < 0.05, ^∗∗^*P* < 0.01, and ^∗∗∗^*P* < 0.001.

The knockdown of SGK1 further increased cellular ROS accumulation and further decreased the ATP content in HK-2 cells under oxidative stress injury (Figures 4(a) and 4(b)). The increased ROS levels and reduced ATP concentration caused by SGK1 knockdown were partly reversed by SB21 (Figures 4(a) and 4(b)). In addition, SB21 did not affect cellular ATP levels (Figure 4(b)); however, it restrained the ROS levels in HK-2 cells under H_2_O_2_ culture conditions (Figure 4(a)). H_2_O_2_ treatment induced a reduction in *ΔΨ*m, which was further decreased after SGK1 inhibition. SB21 obviously increased the H_2_O_2_-induced depolarization of *ΔΨ*m (Figure 5(a)). Moreover, examination of Cytochrome C by confocal microscopy showed that following H_2_O_2_ treatment, Cytochrome C was released into the cytoplasm, as evidenced by the decreased colocalization of Cytochrome C and MitoTracker Red staining in the mitochondria. The released quantity of Cytochrome C was increased by SGK1 knockdown but was significantly decreased in the HK-2 cells after treatment with SB21 compared with the HK-2 cells without SB21 treatment (Figure 5(b)). We then extracted the cytoplasmic and mitochondrial proteins from HK-2 cells and quantified Cytochrome C expression in the cytoplasm and mitochondria separately. Upon SGK1 knockdown, the level of Cytochrome C was increased in the cytoplasm and decreased in the mitochondria. And SB21 could partly decrease the tendency of Cytochrome C to release into the cytoplasm (Figures 5(c)–5(e)). These results indicate that in H_2_O_2_-mediated HK-2 cell injury, SGK1 may contribute to cell survival by improving mitochondrial function via the GSK3*β*-dependent signaling pathway.

### 3.5. SGK1 Promotes Mitochondrial Biogenesis through the GSK3*β* Signaling Pathway

**Figure 6:**
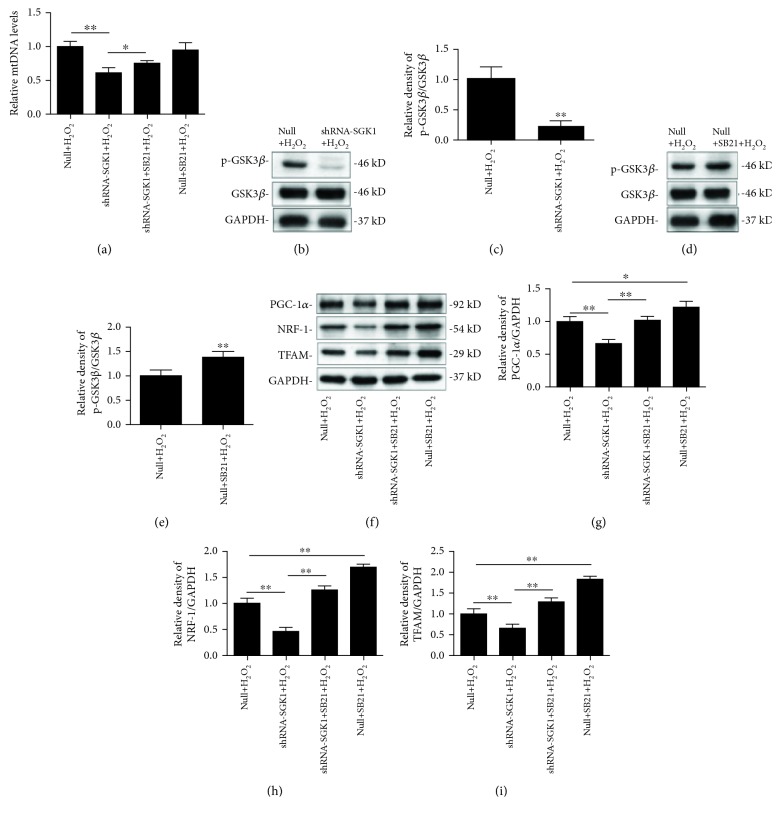
Inhibition of SGK1 downregulates mitochondrial biogenesis in a GSK3*β*-dependent manner. HK-2 cells transfected with scramble control (null) or SGK1 shRNA (shRNA-SGK1) for 72 h were incubated with 10 *μ*M SB21 for 1 h and then treated with H_2_O_2_ (250 *μ*M) for 2 h. (a) The mitochondrial DNA (mtDNA) copy number was detected by real-time quantitative PCR analysis. (b, c) Phosphorylated GSK3*β* protein levels after SGK1 knockdown and (d, e) SB21 treatment are shown. (f–i) Western blot analysis was performed to elucidate the effect of SGK1 knockdown and SB21 treatment on PGC-1*α*, NRF-1, and TFAM protein abundance. Relative protein levels were normalized to GAPDH and total GSK3*β* protein levels. Data are presented as the mean ± SD (*n* = 3). ^∗^*P* < 0.05 and ^∗∗^*P* < 0.01.

Quantitative analysis showed that knockdown of SGK1 dramatically decreased the mtDNA copy number compared with that in the Null+H_2_O_2_ group, and this effect was partly reversed by SB21 exposure (Figure 6(a)). We investigated whether mitochondrial biogenesis is altered in HK-2 cells with SGK1 regulation.

It has been reported that PGC-1*α* and its two downstream target genes, NRF-1 and TFAM, are key regulators of mitochondrial biogenesis [23]. Therefore, we examined the levels of PGC-1*α*, NRF-1, and TFAM in SGK1-knockdown HK-2 cells in the presence or absence of SB21, which indeed effectively phosphorylated and inactivated GSK3*β* (Figures 6(d)–6(e)). We found that the inhibition of SGK1 suppressed the H_2_O_2_-induced phosphorylation of GSK3*β* (Figures 6(b) and 6(c)). Knockdown of SGK1 significantly decreased the expression of PGC-1*α* in H_2_O_2_-stimulated HK-2 cells; however, the effect of SGK1 knockdown on PGC-1*α* abundance was partially blocked by SB21. Treatment with SB21 alone obviously promoted the expression of PGC-1*α*, NRF-1, and TFAM compared with their expression in the Null+H_2_O_2_ group (Figures 6(f)–6(i)). These findings suggest that SGK1 regulates mitochondrial biogenesis through a GSK3*β*-dependent pathway.

## 4. Discussion

In this study, we demonstrated that SGK1 was upregulated and activated in HK-2 cells incubated with H_2_O_2_. SGK1 knockdown aggravated the cell damage and mitochondrial dysfunction caused by H_2_O_2_ exposure, confirming the protective role of SGK1 against oxidative injury. In addition, we showed that the inhibition of GSK3*β* mitigated HK-2 cell injury and partly reversed the damaging effects of SGK1 knockdown upon H_2_O_2_-induced injury, suggesting that SGK1 might protect renal tubular cells by promoting mitochondrial function and the GSK3*β* signaling pathway.

SGK1 promotes cell survival and inhibits cell apoptosis in multiple cell lines, including renal cells [24–26]. SGK1 may represent a specific target to further develop novel therapeutic options to counteract and prevent age-related diseases. SGK1 gene expression is highly dynamic and can be regulated by diverse hormones, cytokines, and external and physiological stimuli, while SGK1 deficiency has been implicated in clinical renal pathologies [27]. SGK1 displays serine/threonine kinase activity and shares structural and functional similarities with the kinases of the Akt family [28, 29]. In this study, we demonstrated that H_2_O_2_-mediated SGK1 activation inhibited HK-2 cell apoptosis and promoted cell survival. In addition, SGK1 is especially more effective under pathophysiological conditions than under basic cellular conditions [30].

The mitochondrial permeability transition pore (MPTP) is located in the inner mitochondrial membrane and plays a critical role in cell death. The MPTP is closed under normal conditions and is triggered and opened when exposed to stressful stimuli, including oxidative stress [31]. Once the pore is opened, any molecule less than 1500 Da can pass through, resulting in mitochondrial swelling, *ΔΨ*m depolarization, and thus ATP depletion, as well as Cytochrome C release into the cytoplasm and initiation of mitochondrial apoptosis [32]. Our data further showed that SGK1 inhibition could exaggerate mitochondrial dysfunction, confirming that SGK1 might inhibit H_2_O_2_-induced cytotoxicity by improving mitochondrial function.

GSK3*β* has gradually been shown to function greatly beyond glycogen metabolism, playing roles in inflammation, immunomodulation, tissue injury, repair, and regeneration [33]. Growing evidence suggests that GSK3*β* plays a detrimental role in AKI [34]. Moreover, GSK3*β* has emerged as the integration point of many significant pathways in renal cells and transfers signals downstream to regulate the opening of the MPTP [35]. A previous study verified that GSK3*β* is dynamically regulated depending on the degree of organ damage [36]. In the present experimental conditions, H_2_O_2_ induced the phosphorylation and inactivation of GSK3*β*.

Because of its wide range of substrates, GSK3*β* plays a key and versatile role in mediating cell viability by regulating mitochondrial function [37, 38]. The N-terminal domain of GSK3*β* has been shown to function as a mitochondrial targeting sequence, permitting the mitochondrial translocation of GSK3*β* in a kinase activity-dependent manner [39]. Dysregulation of the MPTP might be one downstream effect of GSK3*β*, and phosphorylated GSK3*β* increases the MPTP opening threshold [40, 41]. GSK3*β* activation initiates the caspase cascade and results in cell apoptosis. Previous studies have demonstrated that inhibition of GSK3*β* attenuates ceramide-mediated mitochondrial apoptosis by suppressing the caspase pathway [42]. Consistent with these studies, we demonstrated in this study that the GSK3*β* inhibitor SB21 might alleviate HK-2 cell apoptosis by ameliorating oxidative stress and reducing Cytochrome C release into the cytoplasm upon oxidative stress. Moreover, GSK3*β* phosphorylation was related to SGK1 activation, and inhibition of GSK3*β* could alleviate cell apoptosis and mitochondrial dysfunction induced by knockdown of SGK1. These results indicate that the SGK1/GSK3*β* pathway might be activated upon oxidative stress and function as an endogenous antioxidant.

Despite the transient effect on GSK3*β* during the early phase of oxidative stress, inactivation of GSK3*β* promoted the recovery of mitochondrial dysfunction. Consistent with our study, other research demonstrated that pharmacological or genetic inhibition of GSK3*β* diminished mitochondrial Ca^2+^ overload and subsequent cardiomyocyte death but did not modify *ΔΨ*m [43]. Similarly, inactivation of GSK3*β* facilitates the recovery of *ΔΨ*m by suppressing ROS production, leading to cytoprotection from oxidant stress-induced cell death. Interestingly, GSK3*β* inhibition improved the *ΔΨ*m recovery after washout of antimycin A; however, there was no change in *ΔΨ*m during antimycin A treatment [44]. We speculate that the effect of GSK3*β*-based regulation might depend on the duration of GSK3*β* inhibition and specific experimental conditions.

Because mitochondria are involved in various cellular processes, their quantity and quality are both finely tuned. Stimulating mitochondrial biogenesis can restore mitochondrial and renal function [45]. PGC-1*α*, the master regulator of mitochondrial biogenesis, has been shown to be a GSK3*β* substrate [46]. GSK3*β* reduces PGC-1*α* levels by phosphorylating PGC-1*α* and subsequently stimulating PGC-1*α* degradation by the ubiquitin-proteasomal system [47]. Simultaneously, GSK3*β* is a downstream substrate of many signaling pathways, including PI3K/Akt and insulin. Akt was previously shown to affect cell fate by inhibiting the phosphorylation of GSK3*β* [48]. Thus, our results showed that SGK1 may exert renoprotective effects through phosphorylation of GSK3*β* and, consequently, activation of mitochondrial biogenesis. Considering the diversity of the SGK1 downstream substrates, it is explicable that the GSK3*β* inhibitor did not completely block the detrimental effects of SGK1 inhibition on cell apoptosis and mitochondrial function; i.e., other GSK3*β*-independent signaling pathways may contribute to the protective effects of SGK1 [49].

**Figure 7:**
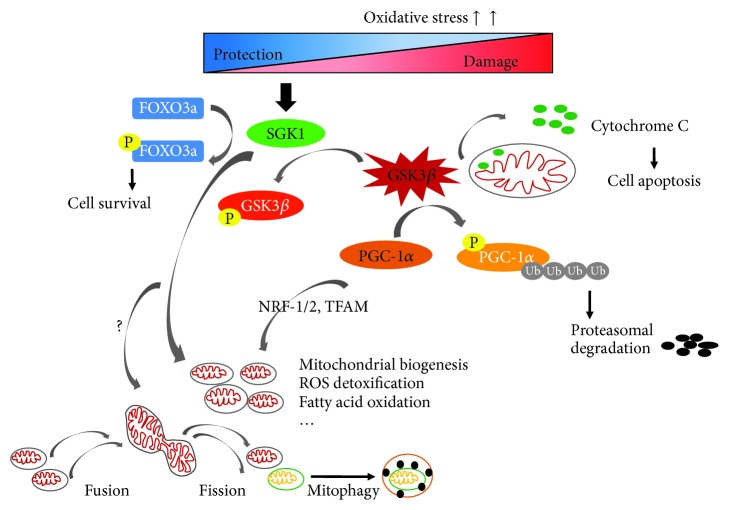
A schematic representation of the proposed mechanisms by which SGK1 regulates mitochondrial function in response to oxidative stress. Oxidative stress dynamically activates SGK1, which in turn protects cell survival through several different pathways. For example, SGK1 may phosphorylate FOXO3a and inhibit cell apoptosis [21] and SGK1 may promote mitochondrial function and mitochondrial biogenesis by phosphorylating and depressing GSK3*β* overactivation, which is widely proven to play dirty in mitochondrial homeostasis. However, whether SGK1 can regulate other processes of mitochondria turnover is worth further study. FOXO3a: forkhead box O3; Ub: ubiquitin; P: phosphorylation.

A balance between mitochondrial autophagy and biogenesis is critical for mitochondrial homeostasis and turnover. With age increasing, mitochondrial integrity and biogenesis declines. Mitochondrial dysfunction is believed to be a major mechanism underlying the process of aging. Evidence suggests that with mitochondrial experimental aggravation speeding up the aging process, in contrast, its experimental amelioration delays the normal aging process [50]. So, the modulation of mitochondrial quality control can be exploited as a therapeutic target against cellular aging. A previous study showed that SGK1 delays the onset of senescence by increasing telomerase activity [20]. Our previous work has shown that SGK1 is dynamically activated during renal ischemia/reperfusion injury in rats and protects renal tubular cells against hypoxia/reoxygenation injury via promoting autophagy [21]. As a form of autophagy, mitophagy is the selective degradation of mitochondria by autophagy. So, it is reasonable to believe that SGK1 may also stimulate mitophagy while promoting autophagy. Therefore, we suggest that SGK1 is a pivotal kinase that regulates the balance between mitochondrial biogenesis and degradation to ensure the effective function of mitochondria. In the future, we will further explore whether SGK1 can activate more specific autophagy, namely, mitophagy, as well as mitochondrial dynamics. As summarized in Figure 7, in the present study, oxidative stress could dynamically regulate SGK1 and SGK1 may act as a physiological protective factor to alleviate renal cell apoptosis, promote the quality control of mitochondria, and therefore serve as a potential intervention target against aging, which is partly dependent on the GSK3*β* signaling pathway.

## Data Availability

The data used to support the findings of this study are available from the corresponding author upon request.
